# Human milk oligosaccharides: bridging the gap in intestinal microbiota between mothers and infants

**DOI:** 10.3389/fcimb.2024.1386421

**Published:** 2025-01-06

**Authors:** Wen Sun, Lin Tao, Chen Qian, Pei-pei Xue, Si-si Du, Ying-na Tao

**Affiliations:** Department of Traditional Chinese Medicine, Shanghai Fourth People’s Hospital Affiliated to Tongji University, Shanghai, China

**Keywords:** human milk oligosaccharides, intestinal microbiota, breast milk, infants, nutrition

## Abstract

Breast milk is an essential source of infant nutrition. It is also a vital determinant of the structure and function of the infant intestinal microbial community, and it connects the mother and infant intestinal microbiota. Human milk oligosaccharides (HMOs) are a critical component in breast milk. HMOs can reach the baby’s colon entirely from milk and become a fermentable substrate for some intestinal microorganisms. HMOs can enhance intestinal mucosal barrier function and affect the intestinal function of the host through immune function, which has a therapeutic effect on specific infant intestinal diseases, such as necrotizing enterocolitis. In addition, changes in infant intestinal microbiota can reflect the maternal intestinal microbiota. HMOs are a link between the maternal intestinal microbiota and infant intestinal microbiota. HMOs affect the intestinal microbiota of infants and are related to the maternal milk microbiota. Through breastfeeding, maternal microbiota and HMOs jointly affect infant intestinal bacteria. Therefore, HMOs positively influence the establishment and balance of the infant microbial community, which is vital to ensure infant intestinal function. Therefore, HMOs can be used as a supplement and alternative therapy for infant intestinal diseases.

## 1 Introduction

The disorder of intestinal flora in early life will seriously affect the health of infants and lead to susceptibility to diseases in later life (Canakis et al., 2020; Underwood et al., 2020). The intestine is the organ with the richest source of symbiotic microbiota in the human body. The symbiotic bacteria residing in the colon account for approximately 70% of all bacteria in the human body, and these bacteria include mainly *Bacteroides, Firmicutes, Actinomycetes* and *Proteobacteria* (Kohl et al., 2018). Living habits, disease factors and dietary factors regulate and affect the composition of the intestinal microbiota (Pickard et al., 2017). Changes in the intestinal microbiota can cause infection, regulate intestinal permeability and intestinal movement, and thus affect host health. The intestinal microbiota can also affect lipid, glucose, and bone metabolism, which are related to conditions such as atherosclerosis, obesity, diabetes and osteoporosis (Patterson et al., 2016; Chen et al., 2021b). The colonization of infant intestinal microbiota is influenced by breast milk, subsequently impacting the development of food allergies in infants (Cukrowska et al., 2020; Wang et al., 2021). Additionally, intestinal microbiota have the capacity to regulate neuroinflammatory responses and mitigate brain injury following neonatal hypoxia and ischemia (Drobyshevsky et al., 2024). Some studies suggest that it is vital to treat these diseases by adjusting the intestinal microbiota and rebuilding the intestinal microenvironment by improving the microbial community structure (Kim et al., 2019; Zhou et al., 2020; Boggio Marzet et al., 2022).

Breast milk is an essential source of infant nutrition and a vital determinant of the structure and function of the intestinal microbial community (Zmora et al., 2019). Human milk oligosaccharides (HMOs) are a crucial component of breast milk (Thum et al., 2021; Poulsen et al., 2022). HMOs can enrich beneficial microbiota, reduce the abundance of pathogenic bacteria, and affect the health status of newborns (Triantis et al., 2018; Sakanaka et al., 2019b; Quinn et al., 2020). HMOs resist human enzymes in the gastrointestinal tract and can reach the infant colon intact and become a fermentable substrate for some intestinal microorganisms (Salli et al., 2019). For example, HMOs could enrich infant gut bifidobacteria and have an antipathogenic effect against *Staphylococcus aureus* (Thomson et al., 2018; Yue et al., 2020). Therefore, HMOs positively affect the establishment and balance of the microbial community, which is vital to ensure normal gastrointestinal function (Strandwitz, 2018). At present, infant formula and food for special medical purposes containing 2’-fucosyl lactose (2’-FL), 3-fucosyl lactose (3-FL), 3-sialyl lactose (3’SL), 6-sialyl lactose (6’SL) and lacto-N- tetrasaccharide (LNT) has been approved by the European Union, the EFSA Panel on Nutrition, Novel Foods and Food Allergens (NDA) (Turck et al., 2022; Turck et al., 2023). The US Food and Drug Administration has evaluated the safety and regulatory of HMOs used in infant formula (Morissette et al., 2023). Meanwhile, HMOs is effective in preventing and treating necrotizing enterocolitis (NEC) (Wang et al., 2024). With the exploration of HMOs, some clinical trials of HMOs on irritable bowel syndrome and other diseases are in progress (Iribarren et al., 2020; Iribarren et al., 2021).

There is a correlation between maternal intestinal and milk microbiota. Some studies have suggested that maternal intestinal microbiota can be transported to milk through the intestinal-mammary route. The baby orally consumes the breastmilk, and microbiota reach spread vertically to the intestine (Zhong et al., 2022). In the first four months after birth, the intestinal flora undergoes significant changes due to the influence of breast milk (Vélez-Ixta et al., 2024). Both 2’-FL and LNnT promote the colonization of Bifidobacterium in infants’ intestines (Berger et al., 2020). Fermentation metabolites derived from HMOs serve not only as substrates for bacterial cross-feeding and as mediators of microbial-host interactions but also possess antibacterial properties (Huertas-Díaz et al., 2023). Recent studies have identified a correlation between HMOs and milk microbiota, potentially linked to the extent of HMOs utilization by microorganisms in the milk (Cheema et al., 2022). Furthermore, the secretor/non-secretor status influences HMOs production, affecting the composition of human milk microbiota and the colonization of infant intestinal flora (Soyyılmaz et al., 2021).

Therefore, this review will describe the structure, influencing factors, and utilization pathways of HMOs, exploring the relationship between maternal gut microbiota and HMOs, with particular emphasis on how HMOs establish a stable infant gut microbiome through breastfeeding. Finally, we will elucidate the extensive potential of various HMOs in regulating microbiota-related diseases in infants based on their current clinical applications.

## 2 Structure of HMOs

HMOs are the third largest solid component of breast milk, following lactose and fat (Thum et al., 2021). The colostrum has a high level of HMOs, and the concentration is 6-15 g/L within one month after delivery. Its concentration decreases with increasing lactation time, and it is 4-6 g/L after 6 months (Thum et al., 2021; Poulsen et al., 2022). HMOs are not digested in the intestines of infants but have essential biological functions. HMOs are considered a bioactive component because of their health benefits to infants (Reverri et al., 2018).

More than 200 HMOs with different structures have been isolated (Peterson and Nagy, 2021). Monosaccharide composition, polymerization rate, charge and acetylation lead to other HMO structures.The oligosaccharides are composed of 3-10 monosaccharides linked by glycosidic bonds to form a straight or branched chain. HMOs are composed of nucleotide sugar molecules linked by glycosidic bonds, and their core structure includes D-glucose (Glc), D-galactose (Gal) and N-acetylglucosamine (GlcNAc), which are further modified by fucose (Fuc) and sialic acid (NeuAc) (Wiciński et al., 2020). HMOs are based on the main chain of lactose (Gal β 1-4LC) as the reducing end, which can be connected by β1-3 or β1-6 and further extended by two disaccharide lactones -N- disaccharide (LNB) or N-acetyllactosamine (Neu5Ac). The backbone structures of complex HMOs generally contain single or repetitive lacto-N-biose (type 1) or lactosamine (type 2) units in either linear or branched chains extending from a lactose core (Hanisch and Kunz, 2021; Yao et al., 2024).

**Figure 1:**
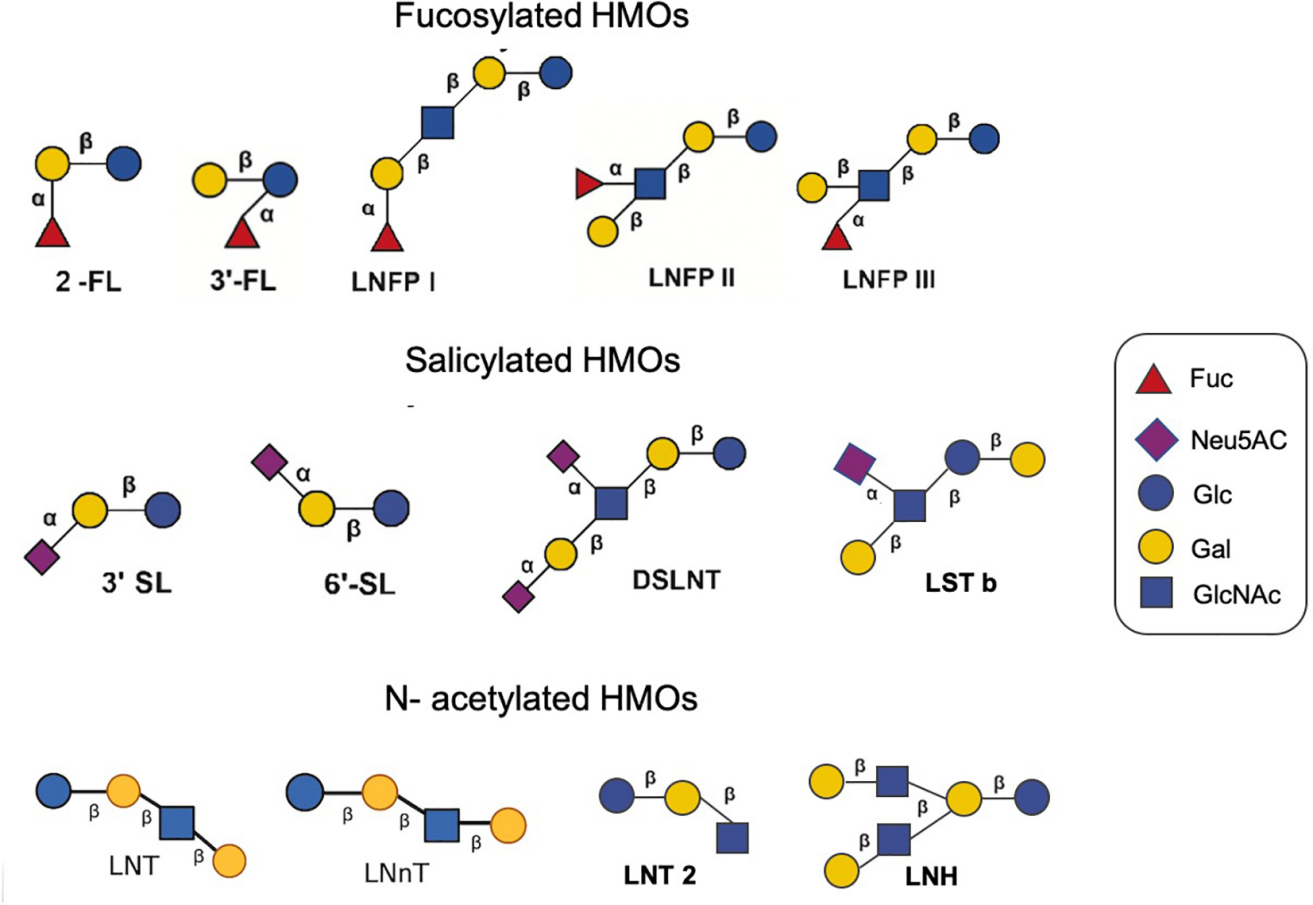
Different structures of several HMOs.

According to the glycosidic bonds of different sugar units, they are mainly divided into fucosylated HMOs, sialylated HMOs, and N- acetylated HMOs (Samuel et al., 2019). Fucosylated HMOs contain 2’-fucosyl lactose (2’-FL), 3-fucosyl lactose (3-FL), lacto-N-fucosyl lactose I (LNFP I), lacto-N-fucosyl lactose II (LNFP II), lacto-N-fucosyl lactose III (LNFP III), etc. Salicylated HMOs include 3-sialyl lactose (3’SL), 6-sialyl lactose (6’SL), disialyllacto-N- tetrasaccharide (DSLNT) and sialyllacto-N- tetrasaccharide (LST), etc. N- acetylated HMOs have lacto-N- tetrasaccharide (LNT), lacto-N-neotetrasaccharide (LNnT), lacto-N- hexaose (LNH) (**Figure 1**). 2’-FL is the most abundant oligosaccharide in breast milk, reaching 4.1 g/L, and is followed by LNT, LNFP I and 3-FL (Thum et al., 2021). The average concentrations of 2’-FL, 3-FL and LNFP I in breast milk are 0.14-2.74 g/L (Thurl et al., 2017), those of LNFP II and LNFP III are 0.21-0.58 g/L (Thurl et al., 2017), that of LNnT is 0.36-1.12 g/L (Ma et al., 2018), that of 3’-SL is 0.19-0.29 g/L (Gidrewicz and Fenton, 2014), and that of DS-LNT is 0.50-0.77 g/L (Van Niekerk et al., 2014). Thurl et al. and Xu et al. found that the ratio between HMOs changed during lactation (Thurl et al., 2017; Xu et al., 2017).

## 3 Factors affecting HMOs

### 3.1 Genetic expression

Genes influence HMO production. The fucosylation of HMOs depends on specific glycosyltransferases in breast cells, including α (Canakis et al., 2020; Underwood et al., 2020)-fucosyltransferase (FUT2) and α1-3/4- fucosyltransferase (FUT3) (Smilowitz et al., 2014; Ewald and Sumner, 2016). The secretion gene (Se) encodes FUT2, and the Lewis blood group gene (Le) encodes the enzyme FUT3 (Soyyılmaz et al., 2021). The difference in gene expression leads to four genetic expression patterns: Se+Le+, Se-Le+, Se+Le- and Se-Le- (**Figure 1**). Glycosylation of the HMO fucoidan is affected by FUT2 and FUT3. Women lacking FUT2 cannot produce 2’-FL or LNFP I (Kunz et al., 2017), and women without FUT3 cannot produce 3’-FL and LNFP II (Thurl et al., 1997). HMO production is regulated by glycosyltransferases in breast cells and is influenced by monosaccharide substrates. These factors may be related to the mother’s physiology, thus slightly affecting the glycosylation process in the initial stage of lactation (Samuel et al., 2019).

### 3.2 The length of lactation

HMO composition is related to the length of lactation (Thurl et al., 2017). The total HMO content in breast milk within one month after delivery is higher than that six months after delivery (Thum et al., 2021; Poulsen et al., 2022). A meta-analysis revealed that the proportion of 6’-SL, LNT and LNnT decreased gradually during lactation, and the proportion of 3’-FL increased gradually during lactation (Zhou et al., 2021).

### 3.3 Maternal diet

The HMO concentration is related to maternal diet. Some studies have shown that different maternal dietary carbohydrates and energy sources have priority in the alterations of HMOs, including fucosylated HMOs (Seferovic et al., 2020). Quin et al. showed that maternal fruit intake was positively correlated with increased HMO absolute abundance (Quin et al., 2020). A cross-sectional study of a cohort of 101 healthy mothers indicated that the diet of secretor mothers had a more significant impact on HMOs than that of nonsecretor mothers. Soluble and insoluble polyphenols and fibers and several insoluble polysaccharides are related to the HMO spectrum of secretors (Selma-Royo et al., 2022).

### 3.4 Maternal body mass index

The HMO concentration is related to the maternal body mass index. A systematic review showed that the content of 2’-FL is increased in mothers with a high body mass index before pregnancy, which is also related to the baby’s weight. The level of sialylated HMOs is higher after premature delivery (Han et al., 2021). Studies have evaluated the maternal HMO content, infant HMO intake, anthropometric measures and body composition in February and June after delivery. The results show that maternal obesity is related to a low concentration of fucoidan and sialylated HMOs, and a change in HMO concentration is related to infant obesity (Saben et al., 2021). Larsson and others propose that maternal body mass index is negatively correlated with 6-SL and positively correlated with 2’-FL and total HMOs (Larsson et al., 2019). Samuel et al. showed that 3′ SL, DSLNT, and 6′ SL levels were higher in low-weight mothers, but the concentrations of LNnT and LNT were lower (Samuel et al., 2019).

### 3.5 Delivery mode and number of deliveries

**Figure 2:**
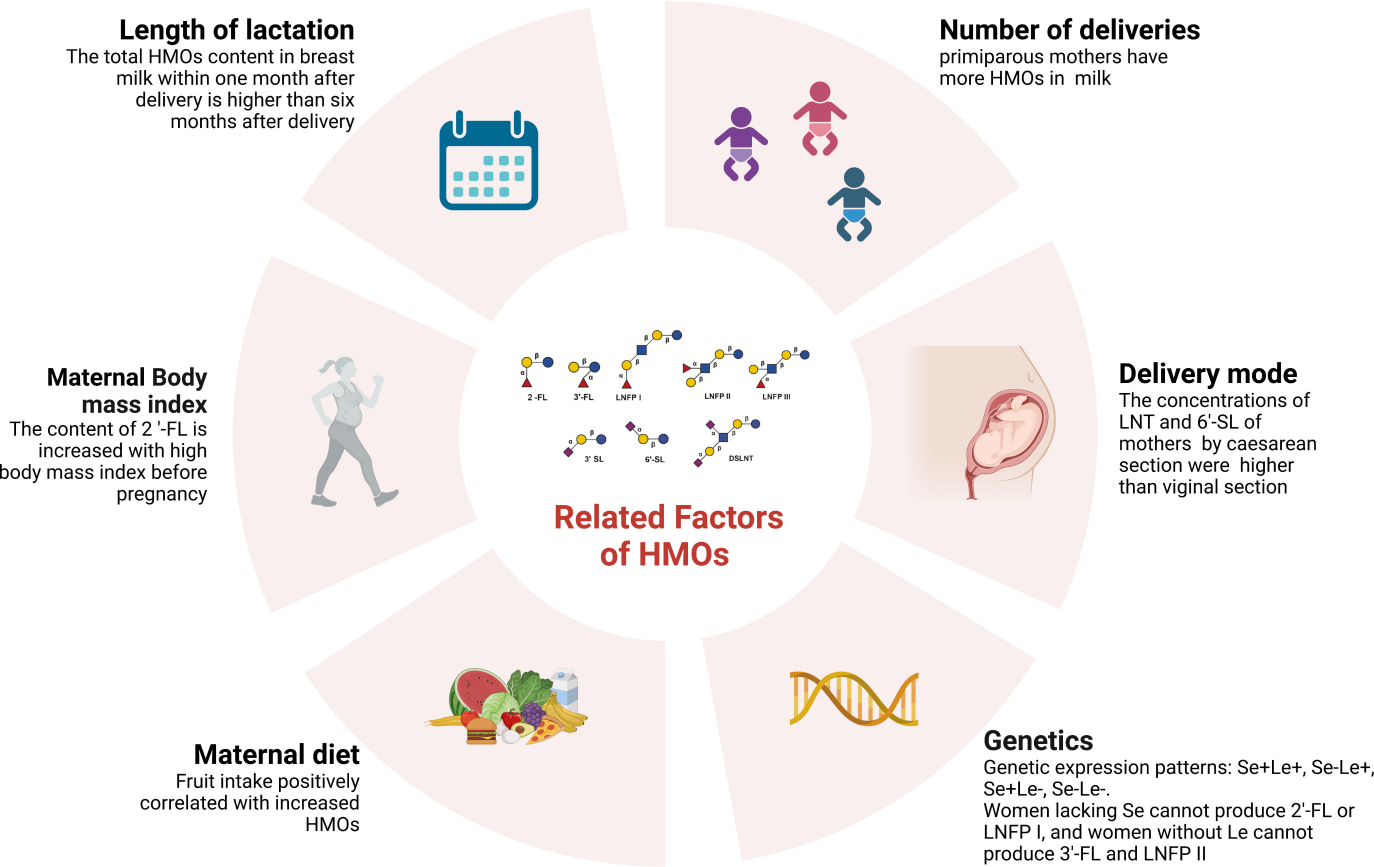
Factors affecting HMOs (Created with BioRender.com).

The concentration of HMOs may also be related to the delivery mode and number of deliveries (Azad et al., 2018; Wang et al., 2020). A study included 290 milk samples from healthy mothers from 7 European countries. It was observed that the concentrations of LNT and 6’-SL of mothers who delivered by caesarean section were higher than those of mothers who delivered vaginally. In comparison, the concentrations of 2’-FL and 3’-SL were lower (Samuel et al., 2019). However, studies have shown no correlation between maternal delivery mode and HMOs (Azad et al., 2018). A study has shown that there are more HMOs in primiparous mothers, among which the concentrations of DSLNT and LNnT are significantly higher, and the total HMO concentration negatively correlates with time (Van Niekerk et al., 2014; Samuel et al., 2019). However, Azad et al. noted that parity increased the total HMO concentration (Azad et al., 2018). Additionally, the concentration of HMOs may be affected by whether the infant was preterm or full-term. However, there is no definite conclusion (Samuel et al., 2019; Jackson et al., 2022) (**Figure 2**).

### 3.6 Other factors

Furthermore, the difference in HMO concentration may be related to different determination methods (Austin and Bénet, 2018; Huang et al., 2019). The usual determination methods include high-performance liquid chromatography−mass spectrometry (HPLCMS), HPLC time-of-flight mass spectrometry, and high-performance anion-exchange chromatography with pulsed amperometric detection (HPAEC-PAD) (Porfirio et al., 2020; Catenza and Donkor, 2021). HPAEC-PAD needs to separate neutral and acidic oligosaccharides in advance (Ruhaak and Lebrilla, 2012). Micellar electrokinetic chromatography (MEKC) is sensitive to Salicylated HMOs (Bao et al., 2007). The sensitivity of capillary electrophoresis (CE) is also limited (Sarkozy et al., 2021). The reference mass spectrometry library of HMOs developed in the laboratory relies on software and databases (Remoroza et al., 2018). Another complicated factor is the lack of analytical standards for the quantitative evaluation of HMO concentrations (Jackson et al., 2022).

## 4 Utilization of HMOs by intestinal microbiota

Some intestinal microbiota can utilize and decompose HMOs. The utilization process of HMOs mainly consists of two parts. HMOs are transported from bacteria extracellularly to intracellularly and hydrolyzed by enzymes.

### 4.1 Bifidobacterium

Most *Bifidobacterium* participate in the metabolism of the HMOs core structure (Ward et al., 2007). The analysis of many sequenced genomes of *Bifidobacterium* showed that many of their codes were related to glycoside hydrolases that are needed to metabolize mucin and HMOs (Zúñiga et al., 2018). *Bifidobacterium* usually needs to remove fucosyl and sialic acid residues when utilizing HMOs. Then, the core of the oligosaccharide is broken down into monosaccharide by different glycosidases (Thongaram et al., 2017). Studies have shown that there are sialidase genes related to the degradation and transport of fucoidan and sialylated HMOs in the genome analysis of *Bifidobacterium*; these genes include oligosaccharide ABC transporter (O'Callaghan et al., 2015; Matsuki et al., 2016), family 1 solute binding proteins (SBPs) and glycosyl hydrolase (Thomson et al., 2018). *Bifidobacterium* can synthesize many oligosaccharide hydrolases, such as fucosidase (GH95 and GH29), β-galactosidase and other glycoside hydrolases, and selectively use HMOs (Verkhnyatskaya et al., 2019). *Bifidobacterium 614* and *418* can utilize 2’-FL, LNDFHI, LNFP I, LNFPII, LNH, LNT and LNnT (Borewicz et al., 2019). *Bifidobacterium longum, infant ATCC 15697* and *infant Bifidobacterium M-63* can utilize 2’-FL and 3’-FL (Kong et al., 2021). *Bifidobacterium* mainly hydrolyses LNT and LNnT through the β-galactosidases Bga42A and Bga2A and releases β-1,3- and β-1,4-linked galactose and LNTII from LNT and LNnT, respectively. LNTII is further catabolized by β-N-acetylglucosaminidase of the GH20 glycosidase family, removing N-acetylglucosaminide GlcNAc and lactose. The latter is hydrolysed into galactose and glucose by β-galactosidase and enters the Leloir pathway, phosphoketolase pathway, amino sugar metabolism pathway, and bifurcation pathway (Yamada et al., 2017). *Bifidobacterium dentium* strains utilize the galactose moiety and release lacto-N-triose from the LNT and LNnT (Moya-Gonzálvez et al., 2021).

### 4.2 Lactobacilli

Most *Lactobacilli* usually have limited ability to utilize HMOs. Nevertheless, *Lacticaseibacillus casei* and *Lacticaseibacillus rhamnosus* contain hydrolases, which rely on the phosphotransferase system to phosphorylate oligosaccharides through a phosphate cascade reaction. Then, they are transported into cells and participate in the metabolism of the HMO core structure (Thongaram et al., 2017). Studies have shown that there are three kinds of α-L-fucosidase (AlfA, AlfB and AlfC) belonging to the GH29 family in Lactobacillus, which can release α 1,6-linked fucose residues from some oligosaccharides (Rodríguez-Díaz et al., 2011). *Lacticaseibacillus casei* transports fucosylation substrates from mannose PTS encoded by the alfEFG gene to cells, and AlfB digests disaccharides in cells into L-fucose and GlcNAc. AlfB and AlfC are active on α-1,3 and α-1,6 bonds in fucosyl-GlcNAc, respectively. AlfC also metabolizes the core fucosylation structure of N-glycosylated protein (Becerra et al., 2020). A sialic acid catabolism nan cluster exists in *Lactobacillus L. sakei 23K* (Zúñiga et al., 2018). Some strains of *Lactobacillus plantarum, Ligilactobacillus salivarius* and *Lacticaseibacillus paracasei* also contain genes for liquid acid metabolism (Hammer et al., 2017). *Lactobacillus acidophilus NCFM* can degrade LNnT (Kong et al., 2020b) most effectively. Thongaram et al. showed that *Lactobacillus acidophilus* grew by using extracellular β-galactosidase encoded by the lacL gene to cleave the terminal galactose of LNnT (Thongaram et al., 2017). *Lacticaseibacillus casei* utilizes LNT II and LNB (Zunga et al., 2021).

### 4.3 Akkermansia

*Akkermansia* can grow in human milk and degrade HMOs. Protein group analysis of *Lactobacillus mucilaginous* showed that the key glycan-degrading enzymes (α-L-fucosidase, β-galactosidase, exoα-sialidase and β-acetylhexosaminidase) could degrade 2’-FL, and LNT could survive in the intestinal tract in early life and settle in the mucous membrane (Kostopoulos et al., 2020). *A. muciniphila* can grow on human milk and is able to degrade HMOs by using glycan-degrading enzymes. These glycan-degrading enzymes hydrolyze HMOs extracellularly into mono- and disaccharides, and then, the liberated sugars are imported into the cell by transporters (Ottman et al., 2017). *A. muciniphila* may use 2′-fucosyllactose, 3′-siallylactose, lacto-N-tetraose, and lacto-N-triose II. *A. muciniphila* is able to survive in the early life environment by consuming oligosaccharides from either breast milk or infant formula (Kostopoulos et al., 2020).

### 4.4 Strains variants

Different strains of probiotics utilize HMOs variably due to genetic differences. For instance, within the HMO utilization gene cluster of *Bifidobacterium longum subsp. infantis*, two main variants exist. Some strains possess a complete set of HMOs utilization genes (H5-positive strains), while H5-negative strains lack the ABC transporters required for the core structure of HMOs. H5-positive strains demonstrate significant growth when exposed to LNnT and LNT. Notably, H5-positive strains are more prevalent than H5-negative strains in breastfed infants (Duar et al., 2020).

## 5 Decomposition and metabolites of HMOs: short-chain fatty acids

The core of oligosaccharides is degraded into monosaccharides and short-chain fatty acids (SCFAs) by different glycosidases. Short-chain fatty acids include acetic, propionic, butyric, and succinic acids (Kong et al., 2021). Acetate and propionate are mainly produced by *Bifidobacterium* spp. and mucin-degrading bacteria such as *Akkermansia muciniphila. Eubacterium hallii* and *Limosilactobacillus reuteri* were able to utilize 1,2-propanediol derived from the fermentation of fucose and rhamnose to produce propionate (Amin et al., 2013; Engels et al., 2016). Butyrate-producing microbiota include bacteria from the families *Ruminococcaceae, Erysipelotrichaceae* and *Lachnospiraceae* as well as bacteria such as *Anaerobutyricum hallii* and *Anaerostipes* spp. (Parada Venegas et al., 2019). The commensal bacterium *Akkermansia muciniphila* also produces propionate from this latter pathway (Belzer et al., 2017). Some bacteria, notably in the *Lachnospiraceae* family, can produce both propionate and butyrate but from different substrates, e.g., *Roseburia inulivorans* (Martin-Gallausiaux et al., 2021).

SCFAs produced by intestinal microbiota can inhibit pathogen replication (Suez et al., 2019), regulate host intestinal immunity, establish immune homeostasis and control intestinal inflammatory diseases (Guzman-Bautista et al., 2020; Porro et al., 2022). Butyrate is the most active SCFA in inhibiting intestinal inflammatory reactions, which helps to protect the intestinal barrier (Leth et al., 2022). Butyrate has been shown to induce colon regulatory T (Treg) cells, which play a major role in preventing local inflammation and regulating the IFN-g and granzyme B gene table of CD8+ T cells (Bachem et al., 2019). Butyrate can induce the expression of ID2 in CD8+ T cells through IL-12 signaling and directly enhance the cytotoxicity of T cells. Valeric acid and butyric acid promote the expression of effector molecules such as IFNγ and TNF (Lu et al., 2022). Acetate promotes the differentiation of Tfh cells (Lu et al., 2022). Butyrate and acetate promote intestinal IgA production through the G protein-coupled receptors GPR41 and GPR43 (Guo et al., 2019). SCFAs, such as indoleacetic acid, can stimulate aromatic hydrocarbon receptors (AHR) (Gutiérrez-Vázquez and Quintana, 2018), mediate anti-inflammatory effects by producing tissue-protective intraepithelial lymphocytes (IELs), and regulate the polarization of innate lymphocytes (ILC) to control intestinal pathogens (Rothhammer and Quintana, 2019). Propionate has been shown to inhibit allergic inflammation in allergic animal models (Trompette et al., 2014).

SCFAs regulate host immunity, the colonization of intestinal pathogens and the expression of their virulence genes, which directly affects the occurrence of diseases. Acetic acid is a key factor in regulating intestinal physiological function and preventing colonization by pathogenic bacteria. It can reduce the pH value of the intestinal tract, has antibacterial effects and inhibits the growth of pathogenic bacteria. Propionate and butyrate inhibited gene expression in Salmonella invasion (Hung et al., 2013). In addition, SCFAs and other organic acids produced during fermentation can be used as cross-utilization nutrients among intestinal bacteria, which may affect the colonization and adhesion of symbiotic bacteria on host epithelial cells (Bianchi et al., 2018).

SCFAs can affect the disease process by regulating metabolism. SCFAs provide energy for intestinal immune cells, such as B cells, memory T cells and effector T cells, by regulating glycolysis, the TCA cycle and β-oxidation (Lu et al., 2022). Colon cells can absorb SCFAs, and the remaining SCFAs can be transported to all body parts through blood. These SCFAs can be used as substrates for synthesizing sugar or lipids and can also be used as cytokines for regulating metabolism (Porro et al., 2022). Butyrate oxidation provides approximately 70% of the energy requirements of colon cells (Leth et al., 2022). Propionates and acetates are necessary for lipogenesis and gluconeogenesis in the liver (He et al., 2020).

## 6 HMOs as a bridge between the intestinal microbiota of mothers and infants

HMOs are produced by the mother and are affected by many factors, including maternal intestinal microbiota. HMOs are related to the maternal intestinal microbiota through milk and affect the infant intestinal microbiota. Therefore, we have reason to believe that HMOs are the link between maternal intestinal microbiota and infant intestinal microbiota. Infants can acquire HMOs through maternal breastfeeding. These HMOs affect the proliferation and adhesion of pathogens in infants and directly reduce the occurrence of intestinal diseases. Furthermore, HMOs can strengthen the intestinal mucosal barrier of infants and regulate their immunity, which can alleviate infant intestinal diseases.

### 6.1 Maternal intestinal microbiota affects human milk microbiota

There is a correlation between maternal intestinal microbiota and milk microbiota. The microbiota in milk may come from maternal breast tissue, maternal intestine, breast skin, or the baby’s mouth. The presence of anaerobic bacteria such as Bifidobacterium in breast milk indicates that the microbiota may be transferred from the mother’s intestine to breast milk (Lyons et al., 2020). There colostrum collected before the first infant lactation contains microbiota, and this indicates that the intestinal-mammary pathway may transport the maternal intestinal microbiota to the lactating mammary gland (Rodríguez, 2014; Damaceno et al., 2017). Using the biomarker strain animal *Bifidobacterium subspecies M8* for metagenomics, a small number of bacteria ingested by the mother could be transported to the baby’s intestine through the oral/intestinal-mammary route (Zhong et al., 2022). This pathway is related to physiological and hormonal changes in the third trimester of pregnancy and the permeability of the intestinal epithelium. Intestinal dendritic cells can penetrate the intestinal epithelium by loosening the tight connection between intestinal epithelial cells and absorbing bacteria from the intestinal cavity. Then, the bacteria are transported to mesenteric lymph nodes by macrophages and finally reach the mammary gland (McGuire and McGuire, 2017). Studies have shown that microorganisms in breast tissue can lead to breast inflammation and breast cancer (Kovács et al., 2020; Marwaha et al., 2020). According to the characteristics of the mammary duct, microorganisms may enter the deep parts of the ducts through the opening of the nipple duct (Spina and Cowin, 2021). Moreover, this method can also affect the microbial community in breast milk. Ultrasonic imaging of nursing mothers demonstrated the retrograde flow of milk from the baby’s mouth due to the baby’s sucking motion, indicating that bacteria can transfer from the baby’s mouth to the mother’s breast (Geddes, 2009). Breastfeeding patterns (direct breastfeeding and breastfeeding with a bottle) are significantly related to the composition of breast milk microbiota, which provides evidence for the retrograde mechanism (Moossavi and Azad, 2020). Milk microorganisms may be derived from breast skin, which contains more Staphylococcus and Corynebacterium (Pannaraj et al., 2017; Demmelmair et al., 2020).

### 6.2 Influence of milk microbiota on infant intestinal microbiota

The microbiota in milk can establish the intestinal microbiota of infants. The changes in infant intestinal microbiota can reflect the maternal intestinal microbiota (Zhang et al., 2022). Studies have shown that most of the intestinal bacteria of babies who are born naturally are breastfed come from the mother’s intestine (Shao et al., 2019). A study conducted a longitudinal analysis of the intestinal microbiota and metabolomics of 70 mothers and infants from the third trimester to their children’s first year. It was found that some mothers’ intestinal microbiota (such as some Bacteroides species) transferred genes to infants’ intestinal microbiota through movable genetic elements to shape the structure and function of the infants’ intestinal microbiota (such as the ability to use HMOs) (Vatanen et al., 2022). Additionally, the results of the microbial analysis show that the composition of microbial groups is related to breastfeeding in babies, indicating that there are bacteria from milk in the baby’s intestines (Banić et al., 2022). Bacterial diversity (Faith species diversity) and composition changes are dose-dependent with the daily breast milk intake ratio (Pannaraj et al., 2017). There is a decrease in *Bacteroides* in infants who are breastfed during the neonatal period (Shao et al., 2019). The diversity and richness of microorganisms in breast milk are the highest in colostrum and gradually decrease with the passage of breastfeeding time. Among these microorganisms, *Staphylococcus, Streptococcus, Acinetobacter, Pseudomonas* and *Lactobacillus* are dominant in colostrum samples (Wan et al., 2020). The regulation of the intestinal microbiota of lactating mothers by diet or probiotics will affect the intestinal microbiota of milk, which may affect the intestinal microbiota of infants (Sindi et al., 2021).

### 6.3 The correlation between milk microbiota and HMOs

Recent studies have shown a relationship between HMOs and milk microbiota that is related to the degree of HMO utilization by milk microorganisms. The relative abundance of milk bacteria is related to the concentration of fucosylation and sialylation HMOs (Cheema et al., 2022). Moossavi et al. analyzed the milk samples of 393 mothers. They found that HMOs were related to the diversity of the microbial community in milk, and *Staphylococcus* was negatively correlated with HMOs (*r*= -0.60 *p*=0.038) (Moossavi et al., 2019). Rubio et al. found that the lower the HMO concentration was, the higher the level of *Staphylococcus* in milk (Cabrera-Rubio et al., 2019). However, some studies have tested healthy breast milk samples and found that the relative abundance of *Staphylococcus* in milk positively correlates with HMO content (Aakko et al., 2017; Williams et al., 2017). The total HMOs in colostrum and transitional milk were positively correlated with the level of *Streptococcus* (Cabrera-Rubio et al., 2019). LNnT and 2’-FL were negatively correlated with *Proteus* and *Actinomycetes* (Gómez-Gallego et al., 2018). In another study, *Streptococcus* and *Staphylococcus* were positively correlated with DFL and LNFPIII (Borewicz et al., 2019).

Rubio et al. found that higher levels of *Lactobacillus* in colostrum and mature milk were related to higher concentrations of 2’-FL. The level of *Lactobacillus* in mature milk is also associated with a higher concentration of LNFP I. Acetylated HMOs were positively correlated with *Lactobacillus, Enterococcus* and *Streptococcus*, while negatively correlated with *Bifidobacterium* (Cabrera-Rubio et al., 2019). Studies have observed that *Bifidobacterium* in milk is positively correlated with sialylated HMOs and LNT and negatively correlated with DS-LNT (Aakko et al., 2017; Moossavi et al., 2019). In addition, there is a positive correlation between fucosylated HMOs and *Akkermansia* (Aakko et al., 2017).

**Figure 3:**
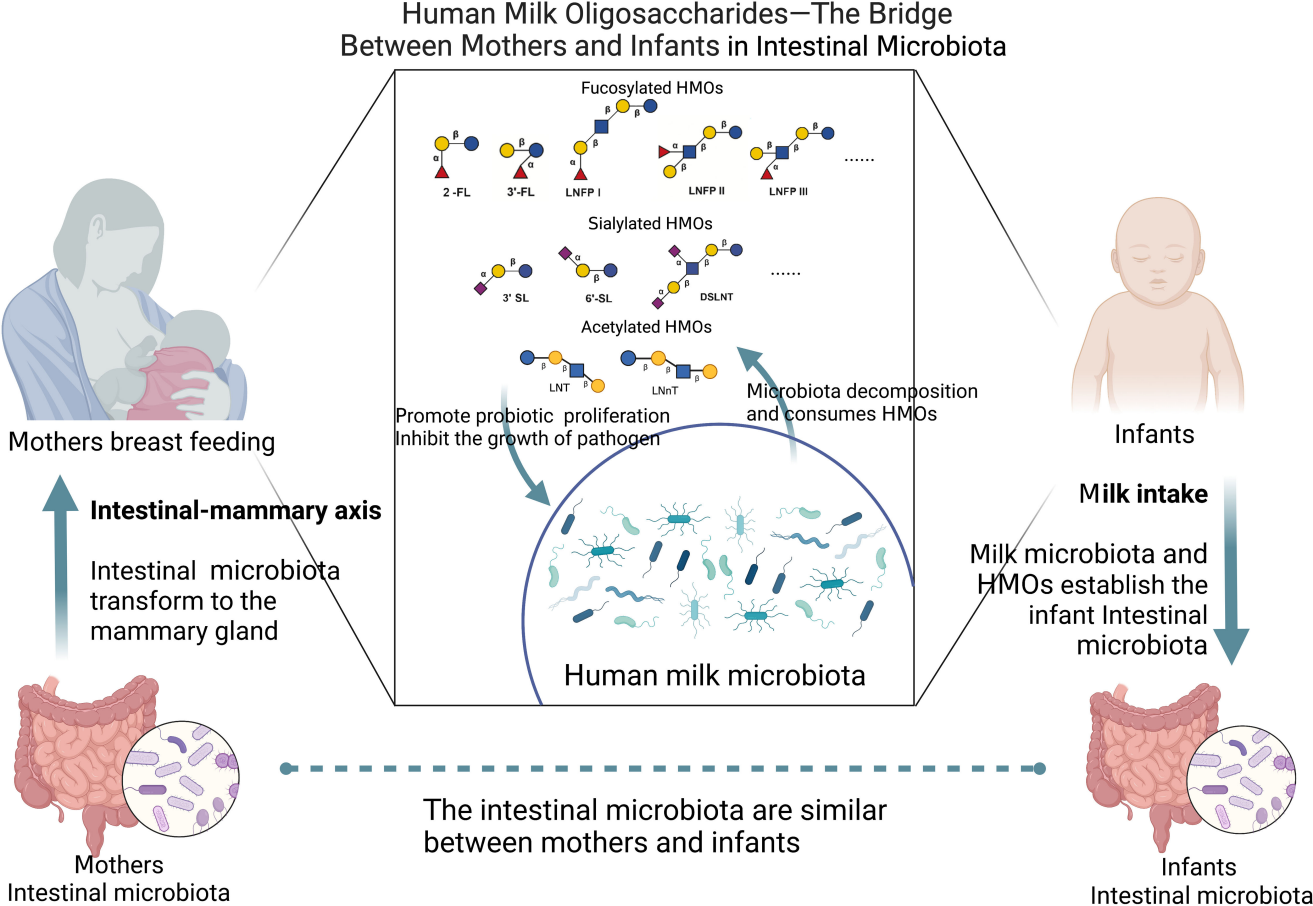
HMOs as a bridge between mothers and infants in intestinal microbiota (Created with BioRender.com).

Therefore, maternal intestinal and milk microbiota, as well as milk microbiota and infant intestinal microbiota, are closely related. Moreover, HMOs are widely present in milk and are connected to milk microbiota. Through maternal lactation, the maternal intestinal microbiota establishes contact with HMOs through milk, and through the effects of the milk microbiota and HMOs on the infant’s intestine, a healthy intestinal microenvironment is established to treat infant intestinal diseases (**Figure 3**).

## 7 HMOs and infant intestinal microbiota

### 7.1 HMOs regulate the growth of intestinal microbiota in infants

#### 7.1.1 Promotion of probiotic growth

At present, some *in vitro* experiments show that HMOs can directly regulate the growth and activity of microbiota. Some studies have used the human intestinal microbial ecosystem simulator (SHIME) and Caco2 cell line to study the effect of HMOs on the adult intestinal microbiota. The results show that 2’-FL and LNnT increase the abundance of *Bifidobacterium* and the content of SCFAs (Natividad et al., 2022). 2’-FL was added to the infant fecal microbiota for culture *in vitro*, and the relative abundance of *Bifidobacterium* was increased as a result (Akkerman et al., 2022; Xu et al., 2022; Dedon et al., 2023). Mass spectrometry analysis of oligosaccharide consumption revealed that *Bifidobacterium longum* and *Bacteroides fragilis* could metabolize fucosylated HMOs efficiently *in vitro* (Marcobal et al., 2010). Single strain culture showed that 2’-FL could promote the growth of *Lactobacillus mucilaginous* isolated from mouse feces (Gao et al., 2022). Studies have shown that *Lacticaseibacillus casei* can grow using LNT as a carbon source (Sakanaka et al., 2019a). After 14 hours of *in vitro* culture, 90.1% of LNT was used in the mixed fecal microbiota of infants, and the richness of *Columbian* and *Bifidobacterium* was explicitly increased. After 36 hours, 60.3% of 3’-FL was used, and *Bacteroides* and *Enterococcus* were specifically enriched (Kong et al., 2021).

Animal experiments *in vivo* show that HMOs can regulate the composition and structure of intestinal microbiota and increase the abundance of probiotics (Xiao et al., 2018). HMOs were added to the humanized mouse model of infant feces transplantation. The 16S rRNA sequencing analysis showed that the abundance of *Bifidobacterium* increased significantly, while that of *Eubacteria* and *Clostridium* decreased (Rubio-Del-Campo et al., 2021). The number of *Bifidobacterium* from the ileum to the colon increased (Mielcarek et al., 2011). HMOs can increase the number of butyrate-producing bacteria, and the ileum mRNA levels of the SCFA receptors Gpr41 (Ffar3), Gpr109 (Hcar2) and Gpr43 (Ffar2) are increased (Jena et al., 2018). 2’-FL can increase the abundance of probiotics, such as *Akkermansia*, in ageing mice (Wang et al., 2022). In the gut-liver-microbiota axis, 2’-FL upregulates acetic acid, propionic acid, butyric acid and total SCFAs by regulating the intestinal microbiota of colitis mice and is positively correlated with *Akkermansia* (Yao et al., 2022).

#### 7.1.2 Effects on the growth of pathogens

The mucin MUC2 mucosal barrier, as the first barrier, prevents direct contact between intestinal bacteria and colon epithelial cells (Yao et al., 2021). If pathogenic bacteria penetrate the epithelial layer and enter the stroma, immune sentinel cells (such as macrophages, dendritic cells and innate lymphocytes) trigger inflammation and the adaptive immune response by activating Th1/Th17 cells (Chen et al., 2021a). In many cases, HMOs can directly inhibit the growth of pathogenic bacteria *in vitro* without relying on host immunity. After HMO treatment, a specific glycosyltransferase gene mutation was identified in the phenotype screen of *group B Streptococcus*, which may be the reason for the antibacterial ability of HMOs (Lin et al., 2017). *Clostridium perfringens* translocation was produced in the intestine of mice that ingested HMOs, and decreased in the ileum and the caecum (Mielcarek et al., 2011). 2’-FL can inhibit the growth of *group B Streptococcus*, such as *Streptococcus agalactiae*, *in vitro* and can regulate the biofilm formation of *Streptococcus agalactiae* (Ackerman et al., 2017). According to the sugar spectrum analysis based on oligosaccharide consumption, *Enterococcus, Streptococcus, Veillonella, Eubacteria, Clostridium* and *Escherichia coli* strains grow poorly in HMOs (Marcobal et al., 2010). 2’-FL and LNnT inhibited the level of *Clostridium difficile* in an intestinal model *in vitro* (Vigsnaes et al., 2021).

HMOs, such as 2’-FL, affect the intestinal microbiota of newborn mice, thus preventing intestinal diseases such as necrotizing colitis (Good et al., 2016; Gart et al., 2022). HMO administration significantly reduced the colonization of enteropathogenic *Escherichia coli* in neonatal mice (Manthey et al., 2014). The combination of HMOs and *Bifidobacterium longum* reduced the *Clostridium* and *G- bacteria* count in mice (Musilova et al., 2017). A study established an infection mouse model of *E. coli O157* and found that ingestion of 2’-FL can reduce the colonization of *E. coli O157* in the intestine of mice by more than 90% (Wang et al., 2020). Intake of 2’-FL reduced the invasion of *Campylobacter jejuni* by 80% in mice (Yu et al., 2016). Transfection of mother mice with the α 1,2-fucosyltransferase gene of the H-2 antigen can inhibit the colonization of *Campylobacter jejuni* in young nursing mice (Ruiz-Palacios et al., 2003). In addition, 3’-SL and 6’-SL can reverse the changes in β diversity of colonic mucosa-associated microorganisms caused by emergency stimulation in mice (Tarr et al., 2015). Therefore, the ability of HMOs to regulate the growth of microbiota directly inhibits the number of pathogenic bacteria and reduces the occurrence of intestinal diseases.

However, several *in vitro* and murine studies indicated that HMOs are directly utilized by either primary degraders or via pathogenic bacteria, including *Shigella. S. typhimurium* accesses fucose and sialic acid within the lumen of the gut in a microbiota-dependent manner. The increase in sialic acid levels induced by microorganisms *in vivo* is helpful for the amplification of *C. difficile* (Ng et al., 2013). The increase in caecal sialidase activity is vital for the growth advantage of *Escherichia coli* (*E. coli*) during intestinal inflammation in mice. The increase in sialidase activity mediates the release of sialic acid in intestinal tissue, which promotes the growth of *E. coli* during inflammation (Huang et al., 2015).

### 7.2 HMOs change the adhesion of intestinal microbiota in infants

#### 7.2.1 Increasing the adhesion of probiotics

HMOs increase the adhesion of probiotics in the intestinal tract, which benefits intestinal health. Studies have shown that 3’-FL and LNT enhance the adhesion of *Lactiplantibacillus plantarum CFS1* 1.85-1.90 times (Kong et al., 2020a). LNT can increase the adhesion of *Lactiplantibacillus plantarum WCFS1* to intestinal epithelial Caco-2 cells (Kong et al., 2021).

#### 7.2.2 Reducing the adhesion of pathogens

HMOs can combine with pathogens in the intestine, reducing the ability of pathogenic bacteria to colonize the intestine, thus reducing its pathogenic effect on the intestine. Many HMOs are soluble receptor analogues of carbohydrates on the surface of epithelial cells, which act as receptor bait. When pathogens combine with them, they reduce their adhesion to intestinal cells and are excreted. *In vitro*, competition tests confirmed that combining HMOs and *Bifidobacterium longum* reduced the *Clostridium* and *G- bacteria* count, which could inhibit potential pathogenic bacteria (Musilova et al., 2017). Yue and other studies show that HMOs can reduce the adhesion of *Staphylococcus aureus* to human colorectal epithelial cells, and the effect is higher than that of oligosaccharides in milk and goat milk (Yue et al., 2020). Manthey et al. showed that HMOs significantly reduced the attachment of enteropathogenic *Escherichia coli* to cultured epithelial cells (Manthey et al., 2014). 2’-FL can combine with *Campylobacter jejuni* to reduce infection (Lane et al., 2011). 2’-FL inhibits the planktonic growth and adhesion characteristics of *Streptococcus mutans*, an oral pathogen associated with dental caries, and reduces the adhesion ability of *Streptococcus mutans CI 2366* and *DSM 20523* to saliva-coated hydroxyapatite (Salli et al., 2020). Colonization of *Campylobacter jejuni* to the human intestinal mucosa *in vitro* was inhibited by fucosylated HMOs (Ruiz-Palacios et al., 2003). Weichert et al. showed that 2’-FL could inhibit the adhesion of *Campylobacter jejuni*, pathogenic *Escherichia coli*, *Salmonella serotype fyris* and *Pseudomonas aeruginosa* to the intestinal human cell line Caco-2 (decreased by 26%, 18%, 12% and 17%, respectively), and 3-FL could reduce the adhesion of pathogenic *Escherichia coli* and *Pseudomonas aeruginosa* (29% and 26%) (Weichert et al., 2013). 2’-FL, 3’-SL and 6’-SL reduced the adhesion and infectivity of human rotavirus (Laucirica et al., 2017). 3’-SL and 6’-SL reduced the adhesion of *Clostridium difficile* to human colon cells, affected the formation of biofilms of *Clostridium difficile*, and decreased the expression of the cell wall protein cwp84, which is related to bacterial adhesion (Piotrowski et al., 2022).

### 7.3 HMOs enhance the intestinal mucosal barrier in infants

#### 7.3.1 Enhancing mucosal barrier function

The intestinal barrier is mainly composed of intestinal mucosal cells and epithelial cells. HMOs increased the transepithelial resistance of Caco-2 cells and decreased the macromolecular permeability of monolayer cells (Wu et al., 2022). HMOs significantly stimulated defensin β-1 (DEFB1) and tight junction protein (ZO-1) in Caco-2 cells (Kong et al., 2020a). Wang et al. showed that HMOs could increase the phosphorylated epidermal growth factor receptor P-EGFR by 58% and decrease P-P38 by 48-89% in Caco-2 cells (Wang et al., 2020). Natividad et al. showed that 2’-FL, LNT, LNnT, 3’-SL and 6’-SL dose-dependently restricted the translocation of fluorescent markers in Caco-2 cells and reduced intestinal permeability (Natividad et al., 2020). Crane et al. showed that HMOs blocked the biochemical action of *Escherichia coli* in the human colon cancer cell line T84, inhibited cGMP of guanylate cyclase activity stimulated by *Escherichia coli* in the T84 cell membrane, and inhibited 125I-STa binding (by 17% and 27%, respectively) (Crane et al., 1994). Some studies have used human intestinal organ chips to study the effect of HMOs on intestinal barrier function. The results showed that the expression levels of the claudin-5 and claudin-8 genes of tight junction proteins increased, while the levels of IL-6 decreased (Šuligoj et al., 2020).

*In vivo* experiments also show that HMOs can enhance the mucosal barrier. When C57BL/C mice with intestinal barrier injury induced by hypoxia ingested HMOs, the number of Ki67-positive cells in the ileum increased by 60-80%, while the number of HIF-1α and caspase-3 in the ileum decreased by 56-71% (Wang et al., 2020). A rat model of necrotizing enterocolitis was treated with HMOs, which directly induced the expression of protein disulfide isomerase (PDI), increased the level of intestinal mucin Muc2, decreased the permeability of the intestine to macromolecular dextran, and reduced bacterial adhesion (Wu et al., 2019). Oral administration of 2’-FL inhibited intestinal permeability in a mouse model of fatty liver disease (Gart et al., 2022). Intake of 2’-FL in mice infected with *Escherichia coli O157* increased the expression levels of the intestinal mucin Muc2 and enhanced intestinal barrier function (Wang et al., 2020). 2’-FL can upregulate endothelial nitric oxide synthase (eNOS) and restore intestinal perfusion in young rats (Good et al., 2016). Oral administration of 2’- FL and 3’-FL to C57BL/6J mice with UC increased the expression of locking protein in colon tissue and reversed the intestinal barrier function of Caco-2 cells and the shortening of colon length (Kim et al., 2023).

#### 7.3.2 Increasing the growth and differentiation of intestinal cells

HMOs can stimulate gastrointestinal maturation. HMOs can induce the differentiation of human intestinal cells and small intestinal epithelial crypt cells, inhibit cell growth and directly regulate the growth cycle of intestinal cells (Kuntz et al., 2008). 3’-SL, 6’-SL and 2 ‘-FL reduced the proliferation of the intestinal epithelial HT-29 and Caco-2Bbe cells, promoted the differentiation of HT-29 and Caco-2Bbe cells, reduced the apoptosis and necrosis of Caco-2Bbe cells (Holscher et al., 2017), and increased the alkaline phosphatase activity of Caco-2Bbe cells (Holscher et al., 2014). Salicylated HMOs can prevent Caco-2 cells from damage by LPS, histamine and chymotrypsin by regulating the cell growth cycle and increasing mitochondrial membrane potential (He-Yang et al., 2020).

HMOs can also increase the differentiation of cells, small intestinal cluster cells and Muc2 cells. Bacterial strains cultured in the feces of infants with dysplasia were implanted into sterile mice and fed a diet containing sialylated oligosaccharides. The results showed that the lean body weight of mice was increased depending on the microbiota (Charbonneau et al., 2016), the level of succinic acid in the caecum was increased depending on sialylated oligosaccharides and microbiota, the number of small intestinal cluster cells was increased, and the signaling pathway of cluster cells induced by succinic acid related to the Th2 immune response was activated (Cowardin et al., 2019). Genome-wide analysis showed that HMOs changed 225 unique target genes related to cell proliferation and differentiation, including the stem cell differentiation marker HMGCS2. After HMO intervention in young rats with necrotizing colitis, the differentiation of Muc2 cells in the crypt-villus axis was enhanced, and the expression of HMGCS2 was upregulated (Li et al., 2020). HMOs can improve the intestinal mucosal barrier and increase the differentiation of intestinal cells. Thus, they can protect the intestine from pathogenic bacteria and alleviate the symptoms of intestinal diseases.

### 7.4 HMOs enhance intestinal immunity in infants

HMOs can reduce intestinal inflammation and regulate intestinal immunity. *In vitro*, HMOs combined with SCFAs induced the development of tolerant dendritic cells (tDCs) and triggered functional regulatory T cells (Xiao et al., 2018). In the inflammatory model of T84 and H4 intestinal epithelial cells invaded by enterotoxigenic *Escherichia coli* type 1 (ETEC) *in vitro*, HMOs reduced the levels of IL-8 that were increased by *Escherichia coli* infection. They also inhibited the transcription and translation of CD14 (He et al., 2016). *In vivo*, HMOs produced specific immune responses by regulating IL-10 and IL-6 (Musilova et al., 2017). Consumption of HMOs by infant C57BL/C mice with intestinal barrier injury induced by hypoxia increased the concentration of IL-8 in serum and the ileum, inhibited the expression of toll-like receptor 4 (TLR4) protein, and activated the nuclear factor κB (NF-κB) pathway in the ileum (Wang et al., 2019). Exosomes containing HMOs are ingested by macrophages responsible for establishing intestinal immunity. Mice pretreated with HMOs encapsulated by exosomes are protected from adhesive invasive *Escherichia coli* infection, and LPS-induced inflammation and intestinal injury are significantly reduced (He et al., 2021).

Fucosylated HMOs, such as 2’-FL, can increase the SCFA content, increase intestinal perfusion, and alleviate the symptoms of intestinal diseases such as necrotizing colitis. 2’-FL attenuates the DC cytokine response *in vitro* in a manner dependent on the AhR receptor (Akkerman et al., 2022). *In vitro* studies showed that 2’-FL promoted the secretion of the mucin MUC2 in intestinal goblet cells through NLRP6, significantly decreased the levels of toll-like receptor 4 (TLR4), myeloid differential protein-88 (MyD88) and nuclear factor κB (NF-κB) in inflammatory cells of LS174T, and inhibited inflammation by inhibiting the TLR4/MyD88/NF-κB pathway (Yao et al., 2023). Intake of 2’-FL in mice infected with *Escherichia coli O157* can reduce intestinal inflammation and increase the content of SCFAs in feces (Wang et al., 2020). 2’-FL can significantly reduce oxidative stress injury and inflammation in the intestine of ageing mice by regulating sirtuin1 (SIRT1)-related and nuclear factor E2-related factor 2 (Nrf2) and increasing the content of SCFAs in the intestine (Wang et al., 2022). Intake of 2’-FL inhibited the release of 60-70% interleukin IL-8, 80-90% IL-1β and 50% neutrophil chemotactic macrophage inflammatory protein 2 (MIP-2) in C57BL/6 mice infected with *Campylobacter jejuni* (Yu et al., 2016).

Salicylated HMOs enhance intestinal immunity and reduce intestinal inflammation. Supplementation of sialylated HMOs can reduce the incidence of necrotizing colitis and the pathological injury score in rats, inhibit the accumulation of mast cells in the ileum of necrotizing colitis rats (He-Yang et al., 2020), reduce the accumulation of IL-1β, IL-6 and TNF-α, reduce mTLR4, NLRP3 and caspase-1 levels in the ileum of rats, restore IκB-α levels in the cytoplasm, and reduce the phosphorylation of NF-κ B P65 in the nucleus (Zhang et al., 2021). Intake of 6’-SL can alleviate the symptoms of intestinal diarrhea in an allergic mouse model and inhibit the number of mast cells in the intestine of mice (Castillo-Courtade et al., 2015). Oral administration of 6’-SL to mice and piglets can reduce necrotizing enterocolitis by reducing apoptosis, inflammation, weight loss and histological appearance, reduce the levels of TLR4-mediated nuclear factor κ light chain enhancer of intestinal NF-kB inflammatory signal, and restore the intestinal barrier of mice (Sodhi et al., 2021; Sodhi et al., 2023). Salivary DS-LNT can improve the survival rate of rats with necrotizing colitis and reduce pathological features (Autran et al., 2016).

**Figure 4:**
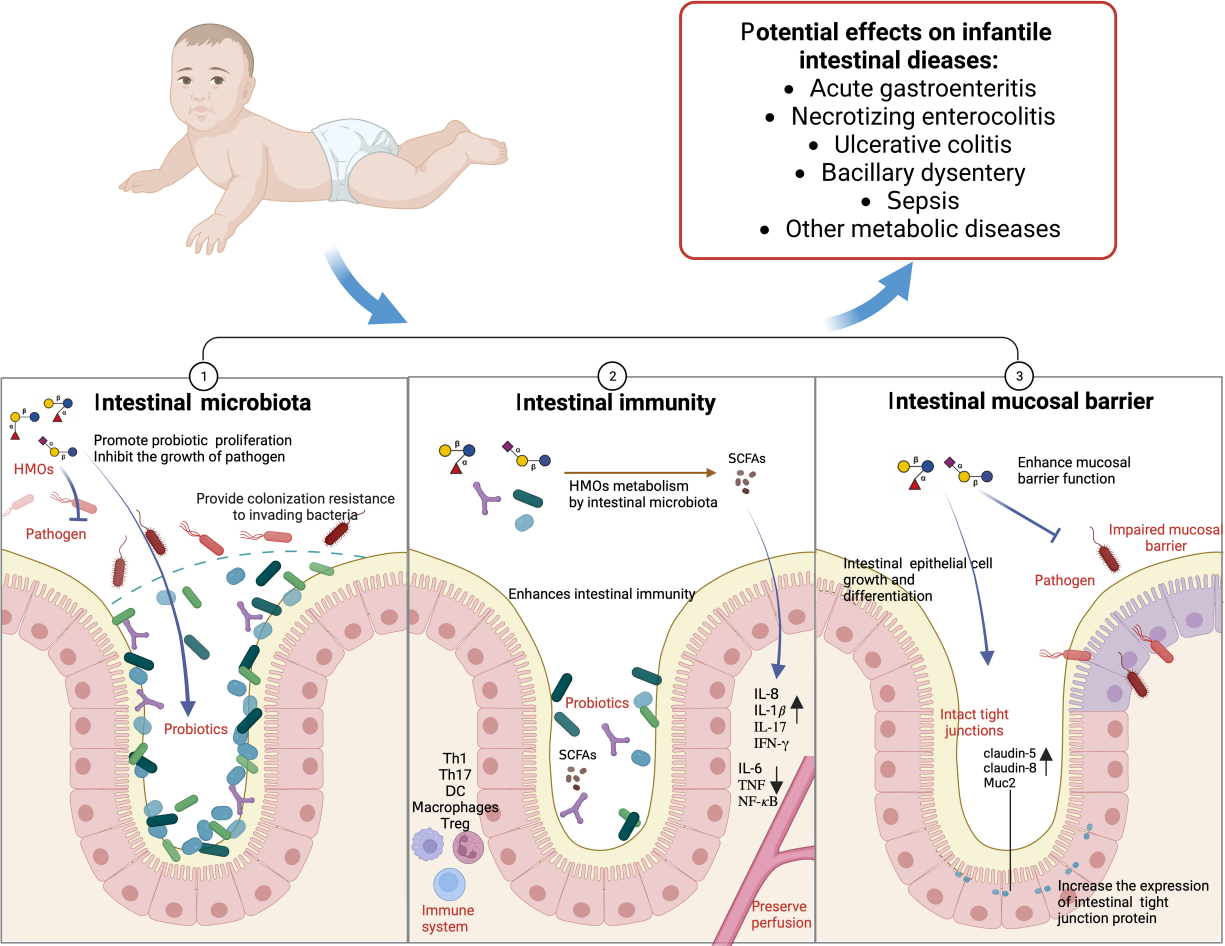
HMOs and infants’ intestinal microbiota (Created with BioRender.com).

In addition, acetyl HMOs, such as LNT, specifically activate GPR35 receptors, relieve colon pain and relieve colitis (Foata et al., 2020). In the immature intestinal epithelial cell line FHs 74, LNnT has a high binding capacity to tumor necrosis factor receptor TNFR1, reducing the intestinal epithelial cell inflammation induced by TNF-α (Cheng et al., 2021). Therefore, HMOs, breast milk, and infant intestinal microbiota have complex interactions (**Figure 4**).

### 7.5 HMOs affect the intestinal activity of infants

HMOs can affect intestinal activity and relieve pain in conditions such as infantile colic or irritable bowel syndrome. In C57BL/6JRj mice with visceral hypersensitivity, the HMO mixture reduced the intestinal distension volume and EMG reaction, promoted most microbial groups and reduced the functional pathways related to visceral hypersensitivity. 2’-FL+DFL and LNT+6’SL decreased the visceral motor response and regulated the serotonin and CGRPα-related pathways (Ferrier et al., 2022).

## 8 The potential of HMOs to influence the infant intestinal microbiota in the intervention of diseases

### 8.1 HMOs increase intestinal probiotics in infants

HMOs can increase the abundance of *Bifidobacterium* in infants’ intestines (**Table 1**). Bode et al. Bode (2012) and Porro et al (2022) proposed that HMOs could affect intestinal *Bifidobacterium*, *Staphylococcus, Streptococcus, Lactobacillus* and *Enterococcus* in infants, and the proliferation of *Bifidobacterium* in infants was correlated with a high HMO content in breast milk (Le Doare et al., 2018). The growth of the intestinal Bifidobacterium community is affected by HMOs and is related to the consumption of various HMOs (Borewicz et al., 2019; Nolan et al., 2020; Nogacka et al., 2021). Studies have shown that Bifidobacterium can utilize 2’-FL, and its abundance negatively correlates with the 2’-FL concentration (Thongaram et al., 2017). The analysis of the intestinal microbiota of infants showed that feeding infants HMOs and the animal *Bifidobacterium* lactis subspecies CNCM I-3446 significantly transformed the subspecies into bifidobacteria (Simeoni et al., 2016). A randomized, double-blind controlled trial also showed that Clostridium in the intestine of infants with 2’-FL was considerably lower than that in infants without 2’-FL, while *Bifidobacterium* was higher than that in infants without 2’-FL (Alliet et al., 2022). A randomized, double-blind controlled trial of overweight children included 75 overweight children aged 6-12 years who were administered 2’-FL and LNnT or glucose placebo orally for 8 weeks. The relative abundance of Bifidobacterium in the experimental group increased significantly after intervention, but no change was observed in the placebo group (Fonvig et al., 2021). From 6 months to 12 months after feeding HMO-containing milk, the relative abundance of *E. coli* and *E. faecalis* in stool samples increased, and the relative level of butyrate increased by 4 times (Nilsen et al., 2020). Consumption of formula containing 2’FL and LNnT increased the levels of acetate and *Bifidobacterium* in infants’ intestines (Dogra et al., 2021).

**Table 1:** Overview of studies used in this review demonstrating the role of HMOs on intestinal in infants (clinical trials).

Study	Workforce	Intervention	Efficiency
Nogacka et al. 173 Prospective clinical trial	Six full-term infant donors of two months of age (three breastfed and three formula-fed)	Independent batch fermentations were performed with feces in 2’fucosyllactose (2’FL) presence. Microbiota composition was analyzed by 16S rRNA gene sequencing at baseline and 24 h of incubation.	Microbiota profiles at baseline were influenced by the mode of feeding and by the intrinsic ability of microbiotas to degrade 2’FL.
Simeoni U et al. 174 Controlled, randomized double-blinded clinical trial	115 healthy full-term infants and a mean age of 5 days. Breastfeed group (n = 39) and the formula supplemented with total oligosaccharide and probiotic (n = 39)	After a 12-week feeding, the study tested the effect of feeding a formula supplemented with galacto-oligosaccharides and 3’-SL and 6’-SL, and the probiotic Bifidobacterium animalis subsp CNCM I-3446. Breastfed infants served as reference group.	In the test group, the probiotic B. lactis increased 100-fold in the stool and was detected in all supplemented infants.
Alliet et al. 175 Double-blind randomized controlled trial	Healthy infants < 14 days old (n = 289) were assigned to a milk-based formula (control group; CG) or the same formula with added 1.0 g/L 2’FL (experimental group; EG).	After 6 months of age, the primary endpoint was weight gain through 4 months of age. Secondary endpoints included gastrointestinal tolerance, stooling characteristics, fecal microbiota.	Weight gain in EG was non-inferior to CG. At 1 month, Clostridioides difficile counts were lower in EG than CG.
Fonvig et al. 176 Randomized, double-blinded, placebo-controlled trial	75 children with overweight (including obesity) ages 6 to 12 years were randomized to receive 2’-FL, a mix of 2’-FL and LNnT (Mix), or a glucose placebo.	Subjects take it orally once per day for 8 weeks. Microbiota composition was analyzed by 16S rRNA gene sequencing.	The relative abundance of bifidobacteria increased after 4 weeks of intervention in the 2’FL-group and Mix-group.
Nilsen et al. 177 Observational clinical trial	Infants at birth (first stool) at 3, 6, and 12-months of age from the general population-based PreventADALL cohort.	The SCFA production and substrate utilization potential of gut microbes were observed by multiomics (shotgun sequencing and proteomics) on infants.	A four-fold increase in relative butyrate levels from 6 to 12 months of infant age.
Dogra et al. 178 Randomized placebo-controlled trial	Healthy, full-term male and female infants from birth to 14 days old (n =103) were randomly assigned to Control or Test group.	Infants were continued feedings with the 2’-FL) and LNnT formulas through 6 months of age. The study used stool microbiota composition, metabolites in several machine-learning-based classification tools.	Among the features of the two groups were the 2-HMO formula higher acetate and Bifidobacterium, as well as lower Carnobacteriaceae members and Escherichia.
Berger et al. 179 Randomized double-blinded controlled multicentric clinical trial	Healthy term infants received either infant formula (control) or the same formula with two HMOs (2’-FL and LNnT; test).	Control and test group from enrollment (0 to 14 days) to 6 months. Breastfed infants (BF) served as a reference group. Stool microbiota at 3 and 12 months, analyzed by 16S rRNA gene sequencing	Marked differences in total microbial abundances. bifidobacteriaceae abundance was higher in test group. There were lower rates of infection-related medication use with HMOs.
Underwood et al. 180 Observational clinical trial	14 mother-premature infant dyads were investigated.	Milk, urine, and stool specimens from infants by mass spectrometry for HMO composition. The stools were analyzed by next-generation sequencing to complement a previous analysis.	Specific HMOs, for example, those associated with secretor mothers, have a protective effect by decreasing pathogens associated with sepsis and necrotizing enterocolitis
Ramani et al. 181 Observational clinical trial	Milk from >50 women Based on the detection of rotavirus in stool samples from the neonates, the infants were classified into 3 groups: symptomatic rotavirus positive (n = 56), asymptomatic rotavirus positive (n = 60), and rotavirus negative (n = 65).	Milk was pooled to account for heterogeneity in HMO composition between different women. Stool samples with gastrointestinal symptoms were screened for rotavirus.	Population studies show the levels of LNT, 2’-FL and 6’-SL correlate with abundance of Enterobacter/Klebsiella in maternal milk and infant stool.

HMOs and probiotics hold significant potential for treating infant diseases. The combination of *Bifidobacterium longum* subsp. *infantis* with HMOs represents a viable strategy to enhance health outcomes for infants and children (Mills et al., 2023; Wong et al., 2024). The inclusion of *Lactobacillus rhamnosus* has been shown to shorten the duration of rectal bleeding in infants with milk protein allergies (Malekiantaghi et al., 2024). *Lactobacillus limousine FPHC2951* has been observed to increase the expression of interleukin-10 (IL-10) mRNA, thereby improving symptoms of colitis (Huang et al., 2024). HMOs may also benefit adolescents with chronic gastrointestinal dysfunction and Autism Spectrum Disorder by modulating the brain-gut axis, potentially improving mood (Aldegheri et al., 2024). Probiotics can produce or metabolize antibacterial substances that inhibit the growth of pathogens or compete with them for nutrients and adhesion to the intestinal epithelium (Kayama et al., 2020; Prete et al., 2020; Huynh et al., 2022). The adhesion of probiotic microorganisms to epithelial cells may trigger a signaling cascade that leads to immune regulation. Additionally, the release of soluble components may directly or indirectly activate immune cells, thus influencing disease progression (Dimidi et al., 2017).

### 8.2 HMOs reduce the risk of intestinal infection in infants

Pathogens are a significant direct cause of intestinal diseases. The microbiota that causes acute gastroenteritis in infants includes pathogenic *Escherichia coli*, *Campylobacter jejuni*, *Salmonella*, and *Clostridium difficile* (Denamur et al., 2021).The incidence of Neonatal necrotizing enterocolitis (NEC) and ulcerative colitis (UC) is related to a decrease in the number of *Bifidobacterium* and *Lactobacillus* and an increase in the levels of *Firmicutes,Bacteroides, Proteus* and *Staphylococcus* (Cui et al., 2021; Moschino et al., 2022). Infantile bacillary dysentery is mainly related to *Shigella* (Qasim et al., 2022). The characterization of intestinal microbiota has potential as a diagnostic and even preventive tool for predicting NEC and can be used as a biomarker for monitoring intestinal diseases (Niemarkt et al., 2019).

Supplementing probiotics can benefit hosts through interactions with intestinal microbiota and immune function (Méndez et al., 2021; Samara et al., 2022), and thus, it has been proposed as a potential tool to prevent Intestinal Infection (Beghetti et al., 2021). There have been clinical trials to regulate and intervene in the intestinal microbiota through probiotic transplantation, fecal microbiota transplantation and gene manipulation to repair the mucosal barrier and treat UC (Glassner et al., 2020). *Escherichia coli Nissle1917* can reduce the intestinal colonization of *Salmonella typhi* by competing for iron intake and secrete microglobulin to limit the growth of adhesion-invasive *Escherichia coli* (Sassone-Corsi et al., 2016). The outer membrane vesicle of *Bacillus polymorpha* inhibited the invasion of *Shigella flexneri*, the expression of the virulence regulator VirF gene and the level of the virulence protein (Xerri and Payne, 2022). *Enterococcus faecalis* expressing antibacterial peptides (bacteriocin 21) replaced indigenous enterococcus colonization and eliminated vancomycin-resistant enterococci (Kommineni et al., 2015). In addition, some specific probiotics may benefit *Helicobacter pylori* gastritis and infantile colic (Vandenplas et al., 2011).

In a randomized, double-blind, controlled multicenter clinical trial, 2’-FL and LNnT were added to infant milk powder and administered for six months. Compared with infants without HMOs, those who did exhibited a significant increase in total microbial abundance. Furthermore, these infants demonstrated a lower risk of infection and reduced antibiotic utilization (Underwood et al., 2015). Mark A et al. sequenced and analyzed infant feces, revealing that HMOs present in secretory mother’s milk decreased the levels of *Proteus* and *Firmicutes* in the infants’ intestines, thereby reducing pathogens associated with sepsis and necrotizing enterocolitis (Berger et al., 2020). Additionally, LNT, 2-’FL and 6’-SL are linked to increased levels of *Enterobacter* and *Klebsiella* in breast milk and infant feces and they may also inhibit the infectivity of neonatal rotavirus (Ramani et al., 2018).

## 9 Conclusion and perspectives

Above all, HMOs can regulate the intestinal microbiota of infants, enhance intestinal mucosal barrier function, affect intestinal function and systemic physiological function of the host through immune function. In addition, HMOs are a link between maternal intestinal microbiota and infant intestinal microbiota. HMOs not only affect the intestinal microbiota of infants but are also related to the maternal milk microbiota. HMOs can influence several intestinal diseases of infants through contact with microbiota, and they have great potential be used as supplementary and alternative therapies for intestinal diseases of infants.

However, some problems remain to be solved. First, the effect of HMOs on the whole body needs further exploration. HMOs can affect the disease process by regulating intestinal microbiota. HMOs have the potential to regulate systemic diseases through intestinal microorganisms.

Secondly, while a correlation exists between maternal intestinal microbiota and HMOs, the specific mechanisms governing HMOs composition remain unclear. Therefore, it is important to explore the synthesis pathways of HMOs in the human body and investigate whether maternal intestinal microbiota influence these pathways.

Finally, as a potential therapeutic drug for intestinal diseases, HMOs need more clinical research. HMO-based clinical trials have been performed for infant and adult intestinal diseases. These studies can provide evidence for the clinical application of HMO-based therapy. However, these studies are usually small in sample size and have poor repeatability. Thus, it is necessary to carry out large-scale multicenter studies to improve the effectiveness of HMOs.

## 10 Literature search methods

Most reviews are based on the research status of HMOs and infant intestinal diseases. The factors influencing HMOs formation and the role of HMOs in the relationship between mother and infant intestinal microbiota are still lacking. We have appraised all forms of published research to ensure that the scientific and clinical implications of the available evidence might be synthesized into useful treatise, which reflects what is known about the intestinal microbiota of mothers and infants and what remains to be elucidated. The literature search was completed up to 20 July 2023 with some updates during the review.

The review was carried out in three stages. First, we described the structure and types of HMOs and explain how organisms use HMOs. Then, we summarized the research on HMOs as a potential treatment for diseases related to the infant intestinal microbiota. Finally, we emphasized the study of HMOs on the relationship between the mother and baby’s intestinal microbiota to illustrate the influence of HMOs on the mother and baby’s microbiota. Preselected keywords, including ‘human milk’, ‘human milk oligosaccharides’, ‘breast’, ‘mammary gland’, ‘microbiota’, ‘infants’, ‘intestinal’, ‘gut’, ‘fucosyllactose’, ‘sialyllactose’, ‘lacto-N-tetraose’, ‘lacto-N-neotetraose’, ‘probiotics’, ‘pathogenic bacteria’, ‘immune’, ‘inflammation’ and ‘gut diseases’, were entered into academic databases and search engines, including PubMed, Medline and Cochrane Library. Although many supporting publications were retrieved, in total, nine human clinical studies (**Table 1**) were included to help clarify our current understanding of HMOs and diseases. Searches were summarized using a PICO framework where population (P) terms were combined with intervention (I) and, in some cases, comparison (C) terms. Outcomes (O) showed the therapeutic effect of HMOs.
